# Inhibitory Effect of 1,8-Cineol on β-Catenin Regulation, WNT11 Expression, and Cellular Progression in HNSCC

**DOI:** 10.3389/fonc.2017.00092

**Published:** 2017-05-22

**Authors:** Anna Roettger, Karl-Ludwig Bruchhage, Maren Drenckhan, Kirsten Ploetze-Martin, Ralph Pries, Barbara Wollenberg

**Affiliations:** ^1^Clinic for Otorhinolaryngology, Head and Neck Surgery, University Clinic Schleswig Holstein, Lübeck, Germany

**Keywords:** HNSCC, 1,8-cineol, WNT, β-catenin, WNT11, glycogen synthase kinase 3

## Abstract

**Objectives:**

Head and neck squamous cell carcinoma (HNSCC) is one of the most common tumors worldwide. The high mortality rates have not changed during the last three decades, and thus there is an enormous need for innovative therapy approaches. Several recent studies suggest an important role of the Wnt/β-catenin signaling pathway in the tumorigenesis of HNSCC. We analyzed the effect of the monoterpene oxide 1,8-cineol on the regulation of the Wnt/β-catenin signaling pathway and the cellular progression of different HNSCC cell lines.

**Methods:**

Permanent HNSCC cell lines were exposed to varying concentrations and times of 1,8-cineol. Regulation and activity profiles of the Wnt/β-catenin signaling cascade were analyzed using Western hybridization experiments, MTT assays, real-time PCR-based epithelial to mesenchymal transition array, and immunohistochemistry.

**Results:**

Exposure of different cell lines to 1,8-cineol treatment resulted in a dose-dependent inhibition of proliferation and a decreased activity of the WNT/β-catenin pathway. We can show the inhibition of glycogen synthase kinase 3 (GSK-3)α/β (Ser-9/21) as well as a corresponding decreased endolysosomal localization, leading to a decreased β-catenin activity. Furthermore, we can show that exposure to cineol functionally results in a reduced expression of WNT11.

**Conclusion:**

In this work, we demonstrate for the first time that 1,8-cineol acts as an inhibitor of the Wnt/β-catenin activity in HNSCC *via* a decreased inhibition of GSK-3, which lead to reduced levels of WNT11 and a dose-dependent decrease of the cellular progression. Our data represent a new mechanism of 1,8-cineol activity, which may lead to novel molecular targets and treatment approaches of this natural drug.

## Introduction

Head and neck squamous cell carcinoma (HNSCC) is one of the most common solid neoplasms worldwide. Its occurrence is being associated with exposure to smoking and alcohol consumption (1). Even though enormous progress concerning the treatment of HNSCC has been made, the mortality rates are still high due to local tumor invasion, development of metastases, and failure of chemo- and radiation therapies (2–6).

Various studies suggest an important role of the Wnt/β-catenin signaling pathway in human tumorigenesis of a number of malignancies, such as melanoma, ovarian cancer, and particularly colorectal cancer (7). Therefore, many proteins of this signaling pathway may serve as promising targets of therapeutic approaches.

In addition, several studies revealed the important role of the Wnt/β-catenin signaling pathway in the immunomodulation of antitumor responses, such as the development and regulation of T-lymphocytes at various stages of thymocyte differentiation. Correspondingly, several recent studies suggest an active role of the Wnt-regulator protein glycogen synthase kinase 3 (GSK-3) in different human cancers, either as a tumor suppressor or as a tumor promoter (8). In HNSCC, the prominent role of GSK-3 in the adenomatous polyposis coli (APC)–β-catenin destruction complex implies that an inhibition of GSK-3 could trigger tumor promotion by activating β-catenin. GSK-3 has, moreover, been shown to be a critical regulator of the nuclear factor (NF) kappaB activity (9, 10). The regulated inhibitory phosphorylation of Ser9GSK-3β is the main cause of WNT activation and has been shown to be upregulated in different epithelial cancers (11). Wnt signaling pathways have established importance in determining tissue development, differentiation, and embryogenesis as well as in tumor progression and invasiveness (12, 13). In colorectal cancer, inactivating mutations in the genes encoding APC or activating mutations of β-catenin result in β-catenin stabilization and tumor progression (14).

There are at least 19 identified Wnt homologs in humans, some of which have been identified as potential signaling molecules in different types of cancer (15). In patients with HNSCC, higher levels of active β-catenin have been shown to correlate with a bad prognosis, suggesting an inhibition of the Wnt/β-catenin cascade as a potential strategy for targeting HNSCC stem-like cells (16).

The monoterpenoid 1,8-cineol is the main component of the essential oil extracted from leaves of *Eucalyptus globulus*. Various biological activities have been attributed to 1,8-cineol, which is commonly applied to treat various chronic and acute inflammatory airway diseases as well as patients with polyposis nasi. Recently, it has been shown that 1,8-cineol leads to decreased activity of the pro-inflammatory NF-κB-signaling by inhibiting its nuclear translocation (17, 18).

Although positive respondings have been reported, the molecular mechanisms of its effect on the cellular progression and recurrence are still not fully understood.

In this work, we analyzed the influence of 1,8-cineol on the regulation of the Wnt/β-catenin signaling pathway in different permanent HNSCC cell lines.

Our data demonstrate for the first time that 1,8-cineol is able to inhibit the Wnt/β-catenin activity in HNSCC and leads to decreased expression levels of WNT11 and a decreased cellular progression and migration rate. Thus, our data indicate a new mechanism of 1,8-cineol activity and may lead to novel treatment approaches of this natural drug.

## Materials and Methods

### Cells and Cell Culture Conditions

The adherent human head and neck squamous cell carcinoma cell lines, such as UT-SCC-16A, UT-SCC-16B, UT-SCC-60A, and UT-SCC-60B, were a generous gift from Reidar Grénmann (University of Turku, Finland). Cells were maintained in Dulbecco’s modified Eagle’s medium (DMEM, 4.5 g/l d-glucose and l-glutamine) supplemented with 10% fetal bovine serum (both Life Technologies, Carlsbad, CA, USA), 1% sodium pyruvate (PAN-Biotech, Aidenbach, Germany), and 1% antibiotics (10,000 U/ml penicillin and 10,000 µg/ml streptomycin) (Biochrom, Berlin, Germany) at 37°C under 5% CO_2_ and 95% air atmosphere.

### Antibodies

Anti-phospho-GSK-3α/β (Ser21/9) (#9331), anti-GAPDH (#2118), and β-tubulin (#2146) were purchased from Cell Signaling Technology (Danvers, MA, USA). Both antibodies were used in a 1:1,000 dilution in TBS containing 3 or 5% BSA (PAA Laboratories, Pasching, Austria). Anti-phospho-GSK-3α/β (Y279/Y216) (#ab68476) and anti-WNT11 (#ab96730) were obtained from Abcam (Cambridge, UK) and used in a 1:1,000 and 1:3,000 dilution in TBS containing 3 or 5% BSA, too. Anti-active-β-catenin (#05-665) was purchased from Merck Millipore (Billerica, MA, USA) and used in a 1:2,000 dilution in TBS and 3 or 5% BSA (Carl Roth, Karlsruhe, Germany).

Anti-Akt (#9272), anti-Akt (Ser473) (#9271), and anti-Akt (Thr308) (#9275) were purchased from Cell Signaling (Billerica, MA, USA) and used in a 1:1,000 dilution in TBS and 3% BSA (Carl Roth, Karlsruhe, Germany). The species specific secondary antibodies anti-mouse IgG (whole molecule)-peroxidase (#A9044) and anti-rabbit IgG (whole molecule)-peroxidase (#A0545) were obtained from Sigma-Aldrich (St. Louis, MO, USA) and used in a 1:50,000 dilution in TBS containing either 5% BSA or 5% dry milk. Dead and apoptotic cells in response to 1,8-cineol were analyzed by annexin V and propidium iodide staining (Apoptosis Kit II; BD, Heidelberg, Germany).

### Western Hybridization

Cells were washed twice in ice-cold PBS and then lysed in RIPA buffer (Cell Signaling Technology, Danvers, MA, USA) containing aprotinin, pepstatin A, protease inhibitor cocktail, and phenylmethanesulfonyl fluoride (Sigma-Aldrich, St. Louis, MO, USA) for 30 min. Three times 15 s ultrasoundbath was used to fragment the cells. Cellular debris were removed by centrifugation (13,000 rpm, 4°C, 10 min). Protein concentration was determined using Bradford assay with Quick Start™ Bradford 1× Dye Reagent (Bio-Rad Laboratories, Hercules, CA, USA). 4× SDS sample buffer was added, and the samples were boiled at 95°C for 5 min. Equivalent amounts of protein (30 µg) were separated by SDS-PAGE in a 10% polyacrylamide gel and transferred onto nitrocellulose membranes by tank blotting. For protein extraction from tissue, the tissue was initially homogenized with 500 µl RIPA buffer and protease inhibitors by using TissueLyser LT (Qiagen, Hilden, Germany) and then incubated and centrifugated as mentioned above.

The membranes were blocked with 0.1% TBS-T containing 5% BSA or 5% dry milk for 1 h at room temperature (RT) and then incubated with the primary antibodies overnight at 4°C. After washing with TBS and 0.1% TBS-T, the membranes were incubated with the species specific secondary antibody for 1 h at RT. The protein bands were visualized using Amersham™ ECL™ Primer Western Blotting Detection Reagent (GE Healthcare, Chalfont St. Giles, Buckinghamshire, UK) and Fusion-FX7 as well as the corresponding software Fusion Bio-1D V12.14 (VilberLourmat, Eberhardzell, Germany), after extensive washing. The nitrocellulose membranes were re-probed overnight at 4°C with GAPDH antibody or β-tubulin antibody in order to determine the loading in each lane. The membranes were washed, incubated with secondary antibody and ECL detection reagent again.

### PCR Array Human Epithelial to Mesenchymal Transition (EMT)

The human EMT RT^2^ Profiler™ PCR Array (Qiagen, Hilden, Germany) profiles the expression of 84 key genes that either change their expression during this process or regulate those gene expression changes. The array includes cell surface receptor, extracellular matrix, and cytoskeletal genes mediating cell adhesion, migration, motility, and morphogenesis; genes controlling cell differentiation, development, growth, and proliferation; as well as signal transduction and transcription factor genes that cause EMT and all of its associated processes.

### Immunofluorescence

An immunohistochemical method was used for the detection of GSK-3α/β (Ser21/9) within the tumor cells. Twenty-four hours prior to treatment with 100 µM cineol, the cells were seeded into 4-well chamber slides (Sarstedt AG; Numbrecht, Germany). After incubation, the medium was removed, and the cells were washed twice with PBS. For the intracellular staining, cells were permeabilized with 0.1% Triton-X-100 (Sigma-Aldrich Chemie GmbH, Steinheim) in PBS. The specific primary antibody anti-phospho-GSK-3α/β (Ser21/9) (#9331) (Cell Signaling, Danvers, MA, USA) was diluted 1:50 with an antibody dilution buffer containing blocking buffer (DCS LabLine, DCS, Hamburg, Germany) and incubated in a humidified chamber for 1 h at RT. After washing with PBS, the secondary antibody goat anti-mouse FITC (Jackson ImmunoResearch Laboratories, West Grove, PA, USA), diluted 1:100 was incubated for 45 min. The cells were rinsed three times in PBS. The nuclei were stained for 1 min using DAPI (1 µg/ml, Roche Diagnostics, Mannheim, Germany). The samples were rinsed three times for 5 min in PBS and were afterward embedded in Fluoromount G (Southern Biotechnologies Associates, Birmingham, AL, USA). Samples were evaluated under a 200 M microscope (Zeiss, Göttingen, Germany) by fluorescence microscopy (AHF Analysentechnik AG, Tübingen, Germany). Cells were photographed using an AxioCam MRm Rev.3 FireWire and the AxioVision Rel. 4.7 software (Zeiss, Jena, Germany).

### 1,8-Cineol

1,8-Cineol was used from Soledum^®^ capsules (Klosterfrau Healthcare Group, Cassella-med GmbH & Co. KG, Cologne, Germany). Native extract (0.6 mg/µl 1,8-cineol) was stored at 4°C, while stock solution was prepared by solving native extract in ethanol (100 mg/ml) followed by final diluting with DMEM high glucose (Biochrom, Berlin, Germany; 1 mg/ml).

### MTT Assay

Cell proliferation was determined by a quantitative colorimetric *3-(4,5-dimethylthiazol-2-yl)-2,5-diphenyl tetrazolium bromide*-*based* (MTT) cell assay. This assay determines viable cell numbers based on the mitochondrial conversion of MTT.

Around 5,000 cells were dispersed into each well of a 96-well plate. Twenty-four hours after culture, four different concentrations of 5-flourouracil or cisplatin were added to the cultures, or the cells were radiated at three doses. After 48 h, 10 µl of MTT dye (5 mg/ml) was added to each well. After 2 h of incubation with MTT, crystals were solubilized and gently shaken for 24 h at RT. The absorbance of the reduced formazan product in control and experimental wells was read using a multi-well ELISA reader at a wavelength of 570 and 690 nm. All growth samples of the MTT assay were carried out in triplicate.

### *In Vitro* Scratch Assay

To analyze cellular *in vitro* migration by using a scratch assay, cells were seeded in cell culture chamber slides and incubated at 37°C under 5% CO_2_ and 95% air atmosphere. When they reached 100% confluence, a straight artificial gap was created in the middle of the chamber. In order to smooth the edges of the scratch and remove floating cells, the cells were washed with medium several times. Afterward the medium was removed and replaced by medium supplemented with 1,8-cineol. Untreated cells were used as negative control. Photographs of the migrating cells were taken every 15 min by a microscope (Keyence, Osaka, Japan) with stage incubator (Tokai Hit, Shizuoka, Japan) as well as multipoint and time lapse function.

## Results

### Dose-Dependent Inhibition of Proliferation of HNSCC by 1,8-Cineol

**Figure 1:**
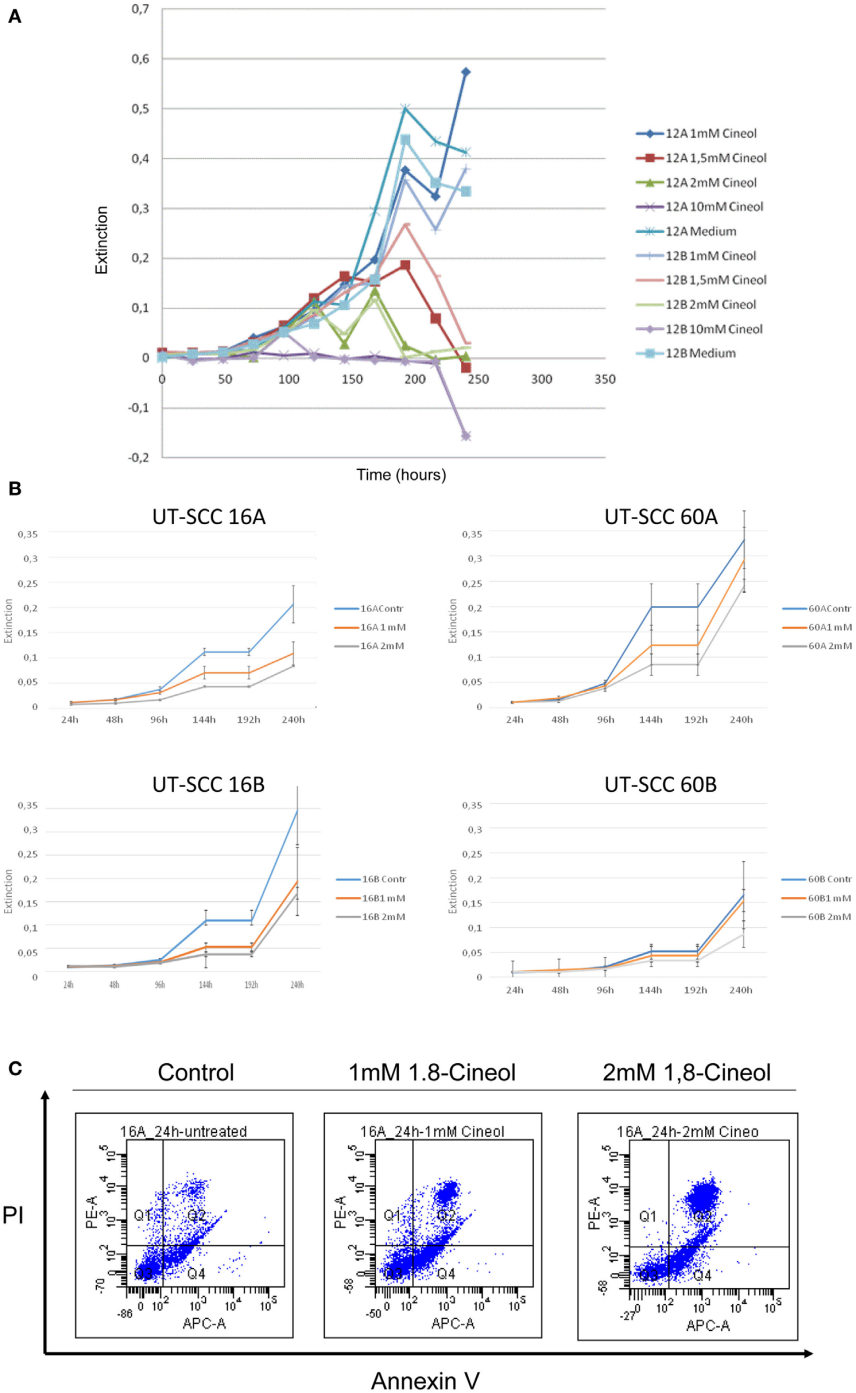
**Dose-dependent reduction of the viability of HNSCC cell lines by 1,8-cineol**. MTT assays were used in order to determine the dose-dependent effects of 1,8-cineol treatment on the cellular viability of permanent HNSCC cell lines. **(A)** Permanent HNSCC cell lines 12A/B were incubated with different concentrations of 1, 1.5, 2, and 10 mM 1,8-cineol, respectively. **(B)** Clearly affected cell growth could as well be observed in the analyzed HNSCC cell 16A/B and 60A/B lines in response to an incubation with 1 and 2 mM 1,8-cineol, respectively. **(C)** HNSCC cell line SCC16A was incubated with 1 and 2 mM 1,8-cineol, and after 24 h of incubation, the cells were harvested and stained with annexin and PI and analyzed by flow cytometry, illustrating the decreased cellular viability in response to 1,8-cineol.

MTT assays were used in order to determine whether 1,8-cineol treatment led to the inhibition of the cellular viability of HNSCC. Concentrations of 1,8-cineol higher than 2 mM resulted in unsolicited strong effects on the cellular viability as well as on the cellular adherence (Figure 1A). Therefore, cell lines UT-SCC60A/B and UT-SCC16A/B were incubated with 1 and 2 mM 1,8-cineol for 240 h, respectively. These cell lines were chosen, since they revealed a prominent WNT/β-catenin signaling in earlier investigations in our group. A strongly decreased proliferation and dose-dependent cellular viability of the analyzed cell lines could be observed in response to treatment with 1,8-cineol (Figure 1B). To underline these data, the HNSCC cell line SCC16A was incubated with 1 and 2 mM 1,8-cineol, and after 24 h of incubation, the cells were harvested and stained with annexin and PI and analyzed by flow cytometry (Figure 1C). In order to carefully analyze the influence of 1,8-cineol on the molecular regulation of the Wnt/β-catenin signaling pathway in preferably vital cells, a maximum dose of 1 mM 1,8-cineol was used for the following investigations.

### Decreased β-Catenin Activity in Response to 1,8-Cineol Treatment

**Figure 2:**
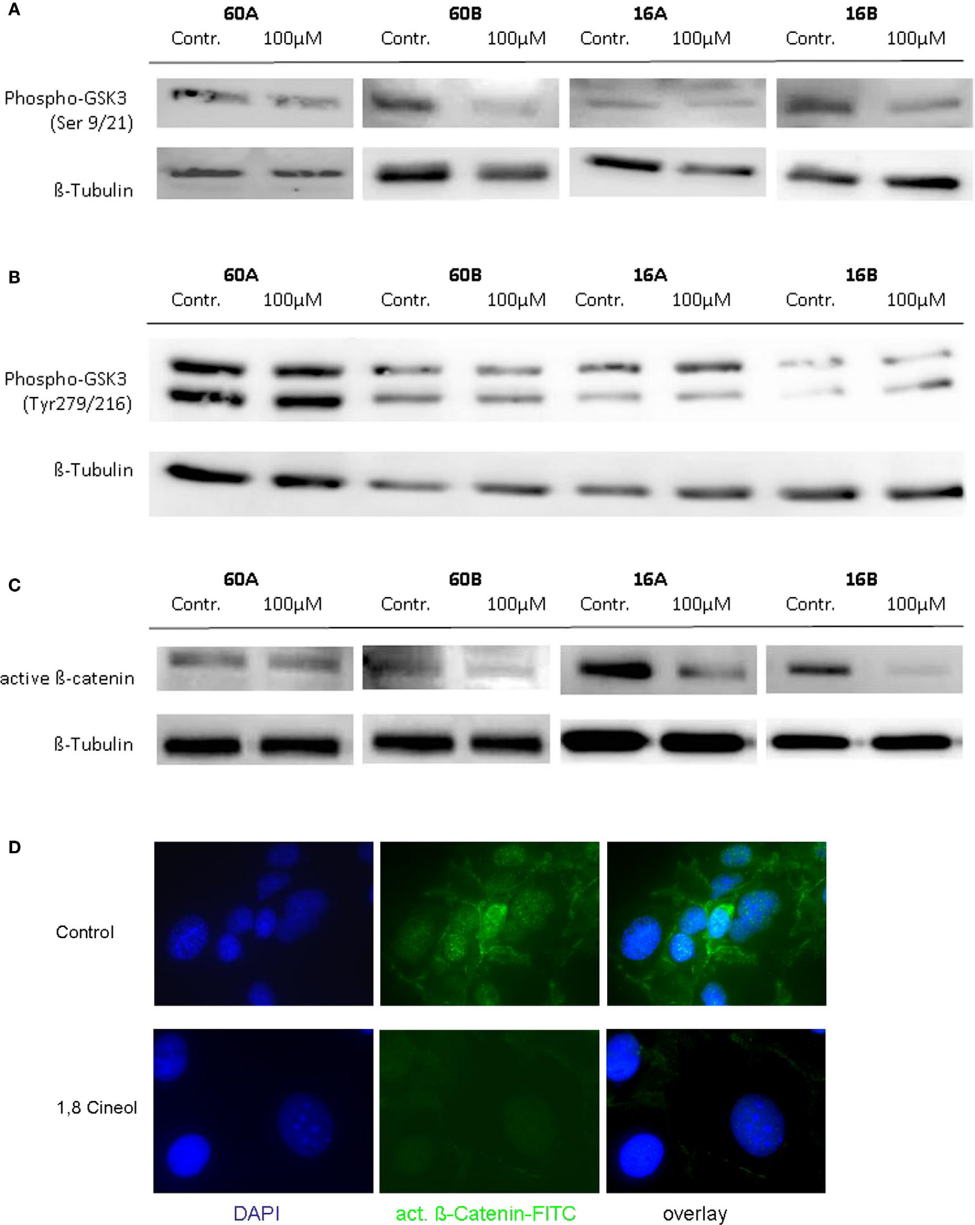
**Expression of active β-catenin and P-glycogen synthase kinase 3 (GSK-3) (Ser9/21) in response to cineol**. Western blot analysis of the whole cell proteins was used to demonstrate the effect of 24 h treatment with 100 µM 1,8-cineol on the protein expression levels of **(A)** phospho-GSK-3 (Ser9/21), **(B)** phospho-GSK-3 (Tyr279/216), and **(C)** active β-catenin, in permanent HNSCC cell lines 60A/B and 16A/B. β-Tubulin was used as a loading control. **(D)** Immunofluorescence staining of active β-catenin in response to 24 h treatment with 100 µM 1,8-cineol. DAPI staining was used to visualize the cell nuclei.

In order to investigate the influence of 1,8-cineol on the activity and regulation of the Wnt/β-catenin signaling cascade, we first analyzed the expression level of active β-catenin, which is the central transcriptional activator protein of this pathway. Our data revealed a strong decrease of active β-catenin in response to 24 h incubation with already 100 µM 1,8-cineol in the analyzed HNSCC cell lines 60B, 16A, and 16B compared to the medium controls, whereas active β-catenin expression was only weakly effected in cell line 60A. These data were corroborated using immunofluorescence staining of β-catenin, indicating an inhibition of the Wnt/β-catenin signaling pathway in HNSCC due to 1,8-cineol treatment (Figure 2).

### GSK-3 Regulation in Response to 1,8-Cineol

To further define the inhibition of the WNT pathway in response to 1,8-cineol, additional investigations were carried out to analyze the corresponding phosphorylation levels of the β-catenin regulator GSK-3. GSK-3 is a constitutively active protein kinase and is inhibited by phosphorylation of GSK-3 on Ser-21 (GSK-3α) or Ser-9 (GSK-3β). Western hybridization experiments were performed to study the expression and phosphorylation levels of GSK-3α/β in the absence and presence of 100 µM 1,8-cineol.

Our data clearly indicate that 1,8-cineol leads to decreased levels of phosphorylated GSK-3α/β (Ser-9/21), which consequently leads to an increased activity of GSK-3 and thus to an increased degradation of β-catenin in the analyzed cell lines (Figure 2B). Phosphorylation of tyrosine-279 in GSK-3α and tyrosine-216 in GSK-3β leads to an enhanced GSK-3 activity. As shown in Figure 1C, expression levels of GSK-3α/β (Tyr-279/216) were not markedly effected by 1,8-cineol (Figure 2C).

### Decreased Endolysosomal Localization of GSK-3 (Ser9/21)

**Figure 3:**
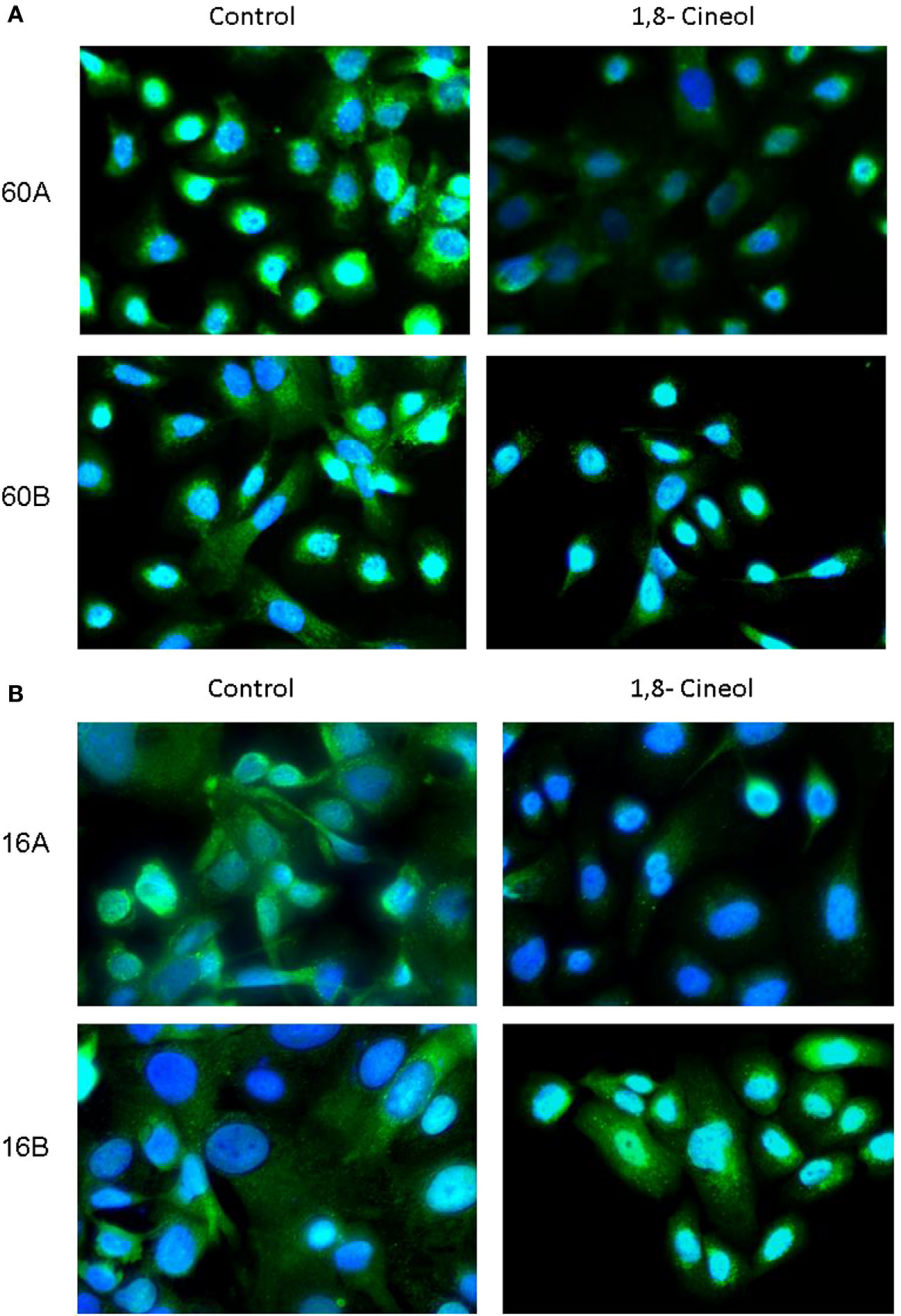
**Subcellular localization of P-synthase kinase 3 (GSK-3) (Ser9/21) in response to cineol**. Immunohistochemical investigations demonstrate a decreased expression and endolysosomal localization of phospho-GSK-3 (Ser9/21) in HNSCC cell lines 60A/B **(A)** and 16A/B **(B)** in response to 100 µM 1,8-cineol compared to the medium control. Nuclei were stained using DAPI.

In addition to its phosphorylation at Ser9/21, GSK-3 activity is also regulated on the level of its subcellular localization. In order to decrease its kinase activity, GSK-3 can be translocated in endolysosomal vesicles to be separated from its substrates. Our immunohistochemical investigations demonstrate a decreased expression as well as a decreased endolysosomal localization of GSK-3 (Ser9/21) in cell lines UT-SCC60A/B and UT-SCC16A/B in response to 100 µM 1,8-cineol, which underlines its decreased inhibition after 1,8-cineol treatment (Figure 3).

### AKT, a Negative Regulator of GSK-3, Is Not Effected by 1,8-Cineol

**Figure 4:**
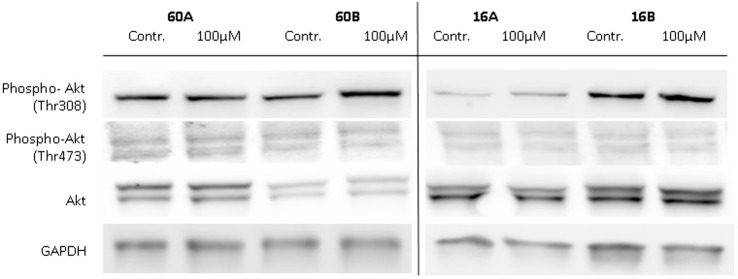
**Expression of Akt, P-Akt (Thr308), and P-Akt (Ser473) in response to 1,8-cineol**. Western blot analysis of the whole cell proteins was used to demonstrate the effect of 100 µM 1,8-cineol on the protein expression levels of Akt, P-Akt (Thr308), and P-Akt (Ser473) in permanent HNSCC cell lines 60A/B and 16A/B. β-Tubulin was used as a loading control.

Glycogen synthase kinase 3 is involved in the regulation of various biosynthetic pathways, and various kinases are able to regulate the GSK-3 activity *via* phosphorylation at its activating or inhibiting phosphorylation sites. Akt kinase (also known as PKB) was identified as an oncogene with serine/threonine kinase activity and was shown to be a negative regulator of GSK-3β activity *in vivo* (19, 20). Akt is activated by phospholipid binding and activation loop phosphorylation at Ser473 and Thr308 by PDK1 (21). Therefore, in a first approach, we analyzed the expression and phosphorylation levels of Akt in response to 100 µM 1,8-cineol. Neither the expression nor the Ser473 and Thr308 phosphorylation levels of Akt were markedly effected in all analyzed cell lines (Figure 4).

### Decreased Wnt 11 Expression in Response to 1,8-Cineol

Since Wnt/β-catenin signaling is well known to be involved in the regulation of the EMT (22), we investigated the transcriptional profiles of various EMT related genes in response to 1,8-cineol. Therefore, we used the PCR-based “RT^2^ EMT Profiler,” which allows the comprehensive analyses of 84 different EMT-related genes. The received data revealed strongly decreased mRNA levels of Wnt11 in response to 100 µM 1,8-cineol in the analyzed HNSCC cell lines.

**Figure 5:**
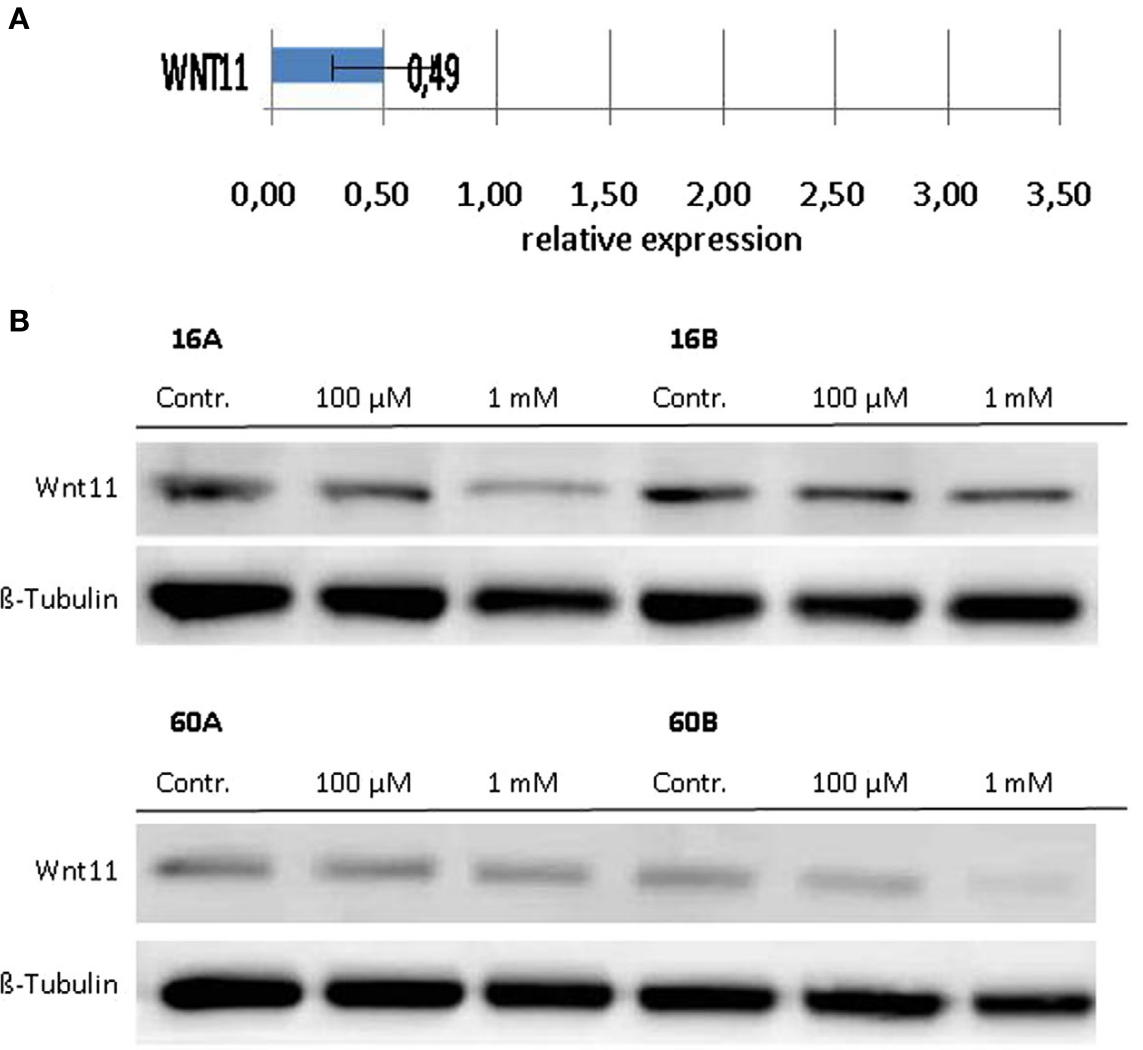
**Expression of WNT11 in response to cineol**. Expression levels of WNT11 were investigated on the **(A)** mRNA as well as on the **(B)** protein level using the RT^2^-PCR Profiler and Western hybridization experiments, respectively. Western blot analysis of the whole cell proteins was used to demonstrate the effect of 1,8-cineol on the protein expression levels in permanent HNSCC cell lines 60A/B and 16A/B. β-Tubulin was used as a loading control.

**Figure 6:**
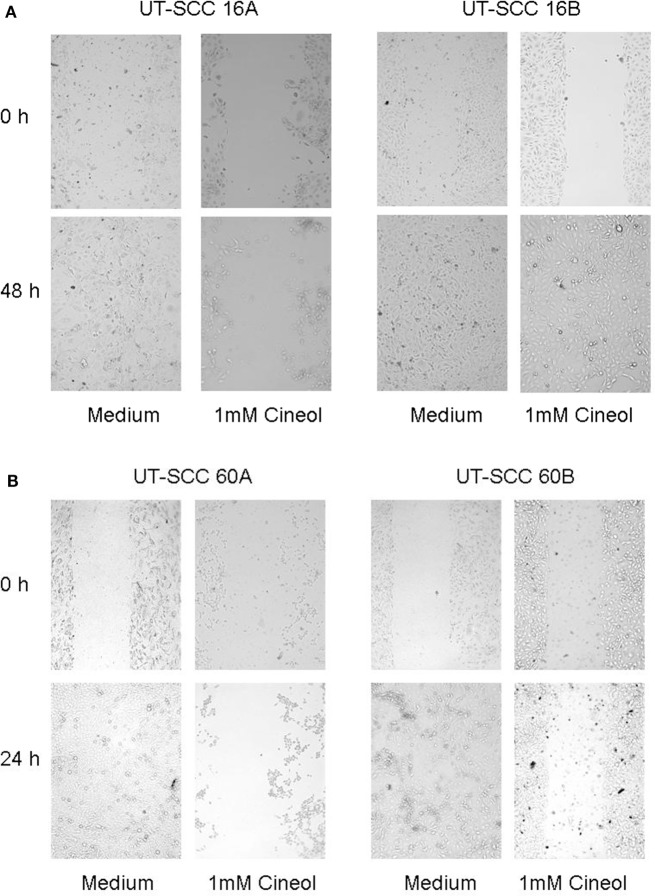
**Decreased migration rate in response to 1,8-cineol**. Scratch assay analyses were carried out, which revealed a decreased migration rate of the analyzed cell lines UT-SCC-16A/B (**A**) and UT-SCC 60A, whereas cell line UT-SCC 60B was not inhibited in response to 1,8-cineol (**B**).

In order to corroborate this highly interesting finding on the protein level, four different HNSCC cell lines were analyzed *via* Western hybridization experiments using a specific anti-Wnt11 antibody. Protein expression of Wnt11 was found to be decreased in response to 1,8-cineol in a dose-dependent manner. All analyzed permanent HNSCC cell lines except cell line UT-SCC 16B showed a strong downregulation of WNT11 expression in response to 24 h treatment with 1 µM cineol (Figure 5). WNT11 has been shown to stimulate the proliferation and migration activity of cancer cells (23). Thus, scratch assay analyses were carried out, which is affected by both proliferation and cellular migration activity. Our data revealed a dose-dependent decreased migration/proliferation rate of the analyzed cell lines in response to 1,8-cineol in cell lines UT-SCC 60A/B and UT-SCC 16A. UT-SCC 16B showed an unaffected migration rate correspondingly to the observed unaffected WNT11 expression in response to 1,8-cineol (Figure 6).

## Discussion

The monoterpene oxide 1,8-cineol resembles the natural plant-derived component of the clinically approved drug Soledum, which is applied for the therapy of airway diseases, such as chronic sinusitis, bronchitis or asthma.

1,8-Cineol has been used as an antibacterial (24) and anti-inflammatory (25, 26) agent. Although clinical trials underline the beneficial effects of 1,8-cineol in treating different types of diseases, the underlying molecular mechanisms remain unclear.

Here, we show that 1,8-cineol is capable to inhibit proliferation of permanent cell lines of head and neck cancer (HNSCC) in a dose-dependent way. These data go along with previously demonstrated antitumor effects against colon cancer, where doses of 1,8-cineol between 5 and 50 mM were used (27). In this work, we used a maximum dose of 1 mM 1,8-cineol in order to carefully analyze the molecular regulation of the Wnt/β-catenin signaling pathway in response to this natural drug in preferably unaffected cells.

One of the major pathways for cell proliferation, dedifferentiation, the immunological structure of the tumor-microenvironment equilibrium and EMT in HNSCC is the WNT pathway. Exposure of HNSCC cell lines to 1,8-cineol leads to specific downregulation of GSK-3α/β (Ser-9/21), a central switch in this pathway. 1,8-Cineol acts specifically, as expression levels of GSK-3α/β (Tyr-279/216) were not markedly affected. In HNSCC, the prominent role of GSK-3 in the APC–β-catenin destruction complex implies that an inhibition of GSK-3 could trigger tumor promotion by activating β-catenin. The regulated inhibitory phosphorylation of Ser9GSK-3β is the main cause of WNT activation and has been shown to be unregulated in different epithelial cancers (11). The downregulation of GSK-3α/β (Ser-9/21) through 1,8-cineol inhibits the inhibitor of this pathway leading to increased activity of GSK-3 and thus to downregulation of active β-catenin, actively blocking the transnuclear signaling of the WNT pathway.

In addition to its phosphorylation at Ser9/21, GSK-3 activity is also regulated on the level of its subcellular localization. In order to decrease its kinase activity, GSK-3 can be translocated in endolysosomal vesicles to be separated from its substrates (28). Our immunohistochemical investigations demonstrate a decreased expression as well as a decreased endolysosomal localization of GSK-3 (Ser9/21) in cell lines UT-SCC60A/B and UT-SCC16A/B in response to 100 µM 1,8-cineol, which underlines its decreased inhibition after 1,8-cineol treatment.

Glycogen synthase kinase 3 is involved in the regulation of various biosynthetic pathways, and various kinases are able to regulate the GSK-3 activity *via* phosphorylation at its activating or inhibiting phosphorylation sites. Akt kinase (also known as PKB) was identified as an oncogene with serine/threonine kinase activity and was shown to be a negative regulator of GSK-3β activity *in vivo* (19, 20). As GSK-3 (Ser9/21) is actively regulated by 1,8-cineol, we attempted to exclude a loop influence on AKT signaling, which we could exclude on the protein level. Various additional cell signaling pathways are known to be involved in the regulation of GSK-3 activity, such as the Wnt-signaling pathway and the MAPK pathway, which may be effected by 1,8-cineol treatment. Traditionally, Wnt signals have been classified into two types, namely, the canonical (β-catenin dependent) and non-canonical (β-catenin independent), respectively. This classification was based on the biological activity, where canonical Wnts (e.g., Wnts 1 and 3a) induced the formation of a secondary axis formation in *Xenopus laevis* embryos and non-canonical Wnts (e.g., Wnts 4, 5a, and 11) did not (29). In fact, Wnt signaling is more complex and known to be context dependent. There are multiple intracellular regulatory cascades, some of which are composed of both canonical and non-canonical components (12, 30).

Dwyer et al. have shown that silencing of β-catenin expression strongly reduced the migratory capacity of breast, prostate, and colon cancer cells *in vitro*. The migratory activity could be attributed to the β-catenin-dependent induction of WNT11, which was shown to be an autocrine regulatory loop. Investigations using conditioned medium harboring recombinant WNT11 identified this protein as the key mediator of migratory activities (31). Our experiments reveal that 1,8-cineol is not only capable to reduce the expression of β-catenin but also of WNT11 in permanent HNSCC cell lines on the transcriptional and protein level. Correspondingly, our data indicate that a decreased expression of WNT11 in response to 1,8-cineol is accompanied with decreased migration rates. In intestinal epithelial cells, it was shown that Wnt11 promotes proliferation and migration by a β-catenin-independent mechanism, which suggests that β-catenin is required for the transcriptional activation of Wnt11 but not for the later cellular activities of the Wnt11 protein (32). Recently, it has been shown that Wnt11 and Wnt5a are capable to inhibit the canonical Wnt pathway *via* a caspase-dependent degradation of AKT (33). Furthermore, gain- and loss-of-function approaches revealed that WNT11 is able to induce an increased activity of matrix metalloproteinase-2 and -9 (23).

To date, 1,8-cineol has been applied to cancer cells already with asthonishingly good results as it was reported to induce apoptosis in leukemia cell lines (34) and colorectal cancer (27). Greiner et al. have demonstrated that 1,8-cineol inhibits the nuclear import of the transcriptional activator protein NF-κB p65 and correspondingly its transcriptional activity (18) leading to decreased cell proliferation. Our findings of the suppressive influence of 1,8-cineol on relevant components in the WNT pathway-driven cancer progression, such as GSK-3 (Ser 9/21), β-catenin, and WNT11, describe a completely novel mode of action, which may lead to novel treatment approaches of this natural drug.

## Author Contributions

AR, K-LB, MD, KP-M, RP, and BW all contributed to the concept and design of the study as well as to data acquisition and interpretation. All authors contributed to writing and revising the manuscript.

## Conflict of Interest Statement

Part of this work was supported by the Klosterfrau GmbH, Köln, Germany, which also provided us with 1,8-cineol for molecular analysis. The study sponsor did not participate in study design and data analysis.

## References

[1] Luce D Guenel P Leclerc A Brugere J Point D Rodriguez J. Alcohol and tobacco consumption in cancer of the mouth, pharynx, and larynx: a study of 316 female patients. Laryngoscope (1988) 98(3):313–6. 10.1288/00005537-198803000-00015 3343882

[2] Leong SP Cady B Jablons DM Garcia-Aguilar J Reintgen D Jakub J Clinical patterns of metastasis. Cancer Metastasis Rev (2006) 25(2):221–32. 10.1007/s10555-006-8502-8 16770534

[3] Chin D Boyle GM Theile DR Parsons PG Coman WB. Molecular introduction to head and neck cancer (HNSCC) carcinogenesis. Br J Plast Surg (2004) 57(7):595–602. 10.1016/j.bjps.2004.06.010 15380692

[4] Douglas WG Tracy E Tan D Yu J Hicks WL Jr Rigual NR Development of head and neck squamous cell carcinoma is associated with altered cytokine responsiveness. Mol Cancer Res (2004) 2(10):585–93. 15498933

[5] Pries R Wollenberg B. Cytokines in head and neck cancer. Cytokine Growth Factor Rev (2006) 17(3):141–6. 10.1016/j.cytogfr.2006.02.001 16540364

[6] Pries R Nitsch S Wollenberg B. Role of cytokines in head and neck squamous cell carcinoma. Expert Rev Anticancer Ther (2006) 6(9):1195–203. 10.1586/14737140.6.9.1195 17020454

[7] Polakis P. The many ways of Wnt in cancer. Curr Opin Genet Dev (2007) 17(1):45–51. 10.1016/j.gde.2006.12.007 17208432

[8] Patel S Woodgett J. Glycogen synthase kinase-3 and cancer: good cop, bad cop? Cancer Cell (2008) 14(5):351–3. 10.1016/j.ccr.2008.10.013 18977324 PMC3006450

[9] Force T Woodgett JR. Unique and overlapping functions of GSK-3 isoforms in cell differentiation and proliferation and cardiovascular development. J Biol Chem (2009) 284(15):9643–7. 10.1074/jbc.R800077200 19064989 PMC2665084

[10] Mishra R. Glycogen synthase kinase 3 beta: can it be a target for oral cancer. Mol Cancer (2010) 9:144. 10.1186/1476-4598-9-144 20537194 PMC2906469

[11] Kockeritz L Doble B Patel S Woodgett JR Glycogen synthase kinase-3 – an overview of an over-achieving protein kinase. Curr Drug Targets (2006) 7(11):1377–88. 10.2174/1389450110607011377 17100578

[12] Clevers H Loh KM Nusse R. Stem cell signaling. An integral program for tissue renewal and regeneration: Wnt signaling and stem cell control. Science (2014) 346(6205):1248012. 10.1126/science.1248012 25278615

[13] Niehrs C. The complex world of WNT receptor signalling. Nat Rev Mol Cell Biol (2012) 13(12):767–79. 10.1038/nrm3470 23151663

[14] Najdi R Holcombe RF Waterman ML. Wnt signaling and colon carcinogenesis: beyond APC. J Carcinog (2011) 10:5. 10.4103/1477-3163.78111 21483657 PMC3072659

[15] Anastas JN Moon RT WNT signalling pathways as therapeutic targets in cancer. Nat Rev Cancer (2013) 13(1):11–26. 10.1038/nrc3419 23258168

[16] Lee SH Koo BS Kim JM Huang S Rho YS Bae WJ Wnt/beta-catenin signalling maintains self-renewal and tumourigenicity of head and neck squamous cell carcinoma stem-like cells by activating Oct4. J Pathol (2014) 234(1):99–107. 10.1002/path.4383 24871033

[17] Muller J Greiner JF Zeuner M Brotzmann V Schafermann J Wieters F 1,8-cineole potentiates IRF3-mediated antiviral response in human stem cells and an ex vivo model of rhinosinusitis. Clin Sci (Lond) (2016) 130(15):1339–52. 10.1042/CS20160218 27129189

[18] Greiner JF Muller J Zeuner MT Hauser S Seidel T Klenke C 1,8-Cineol inhibits nuclear translocation of NF-kappaB p65 and NF-kappaB-dependent transcriptional activity. Biochim Biophys Acta (2013) 1833(12):2866–78. 10.1016/j.bbamcr.2013.07.001 23872422

[19] Hemmings BA Akt signaling: linking membrane events to life and death decisions. Science (1997) 275(5300):628–30. 10.1126/science.275.5300.628 9019819

[20] Kennedy SG Wagner AJ Conzen SD Jordan J Bellacosa A Tsichlis PN The PI 3-kinase/Akt signaling pathway delivers an anti-apoptotic signal. Genes Dev (1997) 11(6):701–13. 10.1101/gad.11.6.701 9087425

[21] Alessi DR Andjelkovic M Caudwell B Cron P Morrice N Cohen P Mechanism of activation of protein kinase B by insulin and IGF-1. EMBO J (1996) 15(23):6541–51. 8978681 PMC452479

[22] Lamouille S Xu J Derynck R. Molecular mechanisms of epithelial-mesenchymal transition. Nat Rev Mol Cell Biol (2014) 15(3):178–96. 10.1038/nrm3758 24556840 PMC4240281

[23] Mori H Yao Y Learman BS Kurozumi K Ishida J Ramakrishnan SK Induction of WNT11 by hypoxia and hypoxia-inducible factor-1alpha regulates cell proliferation, migration and invasion. Sci Rep (2016) 6:21520 10.1038/srep21520 26861754 PMC4748282

[24] Giamakis A Kretsi O Chinou I Spyropoulos CG. Eucalyptus camaldulensis: volatiles from immature flowers and high production of 1,8-cineole and beta-pinene by in vitro cultures. Phytochemistry (2001) 58(2):351–5. 10.1016/S0031-9422(01)00193-5 11551563

[25] Juergens UR Stober M Schmidt-Schilling L Kleuver T Vetter H. Antiinflammatory effects of euclyptol (1.8-cineole) in bronchial asthma: inhibition of arachidonic acid metabolism in human blood monocytes ex vivo. Eur J Med Res (1998) 3(9):407–12. 9737886

[26] Juergens UR Stober M Vetter H. Inhibition of cytokine production and arachidonic acid metabolism by eucalyptol (1.8-cineole) in human blood monocytes in vitro. Eur J Med Res (1998) 3(11):508–10. 9810029

[27] Murata S Shiragami R Kosugi C Tezuka T Yamazaki M Hirano A Antitumor effect of 1, 8-cineole against colon cancer. Oncol Rep (2013) 30(6):2647–52. 10.3892/or.2013.2763 24085263

[28] Kim H Vick P Hedtke J Ploper D De Robertis EM. Wnt signaling translocates Lys48-linked polyubiquitinated proteins to the lysosomal pathway. Cell Rep (2015) 11(8):1151–9. 10.1016/j.celrep.2015.04.048 26004177

[29] Kawano Y Kypta R. Secreted antagonists of the Wnt signalling pathway. J Cell Sci (2003) 116(Pt 13):2627–34. 10.1242/jcs.00623 12775774

[30] Clevers H Nusse R Wnt/beta-catenin signaling and disease. Cell (2012) 149(6):1192–205. 10.1016/j.cell.2012.05.012 22682243

[31] Dwyer MA Joseph JD Wade HE Eaton ML Kunder RS Kazmin D WNT11 expression is induced by estrogen-related receptor alpha and beta-catenin and acts in an autocrine manner to increase cancer cell migration. Cancer Res (2010) 70(22):9298–308. 10.1158/0008-5472.CAN-10-0226 20870744 PMC2982857

[32] Ouko L Ziegler TR Gu LH Eisenberg LM Yang VW. Wnt11 signaling promotes proliferation, transformation, and migration of IEC6 intestinal epithelial cells. J Biol Chem (2004) 279(25):26707–15. 10.1074/jbc.M402877200 15084607 PMC1351009

[33] Bisson JA Mills B Paul Helt JC Zwaka TP Cohen ED. Wnt5a and Wnt11 inhibit the canonical Wnt pathway and promote cardiac progenitor development via the Caspase-dependent degradation of AKT. Dev Biol (2015) 398(1):80–96. 10.1016/j.ydbio.2014.11.015 25482987

[34] Moteki H Hibasami H Yamada Y Katsuzaki H Imai K Komiya T. Specific induction of apoptosis by 1,8-cineole in two human leukemia cell lines, but not a in human stomach cancer cell line. Oncol Rep (2002) 9(4):757–60. 12066204

